# Complication of osteo reconstruction by utilizing free vascularized fibular bone graft

**DOI:** 10.1186/s12893-020-00875-9

**Published:** 2020-10-02

**Authors:** Qifeng Ou, Panfeng Wu, Zhengbing Zhou, Ding Pan, Ju-yu Tang

**Affiliations:** Department of Orthopedics, Hand and Microsurgery, Xiangya Hospital, Central South University, No.87 XiangYa Road, Changsha, 410008 Hunan China

**Keywords:** Free vascularized fibular graft, Extremities, Spine, Mandible, Fibular epiphyseal transfer, Osteo reconstruction, Complication

## Abstract

The success of free vascularized fibular bone graft (FVFBG) has accelerated the osteo reconstruction which results from trauma, resection of a tumor or an infectious bone segment, or correction of congenital deformity. But the complication behind should not be overlooked. The failure could necessitate a second surgery, which prolong the rehabilitation period and produce further health cost. Worst, the patients may suffer a permanent impaired ankle function, or a sustained morpho-functional loss on reconstructive area which are hard to save. To provide an overview of the complication related to reconstruction by FVFBG, a narrative review is conducted to identify the complications including their types and rates, the contributing factors, the approaches to measure and the techniques to avoid. Methodologically, by quick research on Pubmed and abstract reading of reviews, we characterize five reconstructive areas where FVFBG were most frequently applied: extremities, mandible, spine, osteonecrosis of femoral head, and penile. Following, the complications on different reconstructive areas are retrieved, studied and presented in five (or more specifically, six) separate sections. By the way, meaningful difference between FVFBG and other bone flap was presented in a few words if necessary. Donor-site morbidities were studied and summarized as a whole. In these literatures, the evidences documented on limb and mandibular reconstruction have the fullest detail, followed by the spine and lastly the penile. In conclusion, FVFBG, though a mature technique, needs further deep and comprehensive study and maybe device-based assistance to achieve better reconstructive effect and minimize donor-site damage.

## Background

Since 1986, the free vascularized fibular bone graft (FVFBG) has been increasingly applied in restoration of bone defect because of trauma, tumors, infection or congenital anomalies. Due to its integration potential and abundant blood supply, it also has a varied utilization in spinal fusion and osteonecrosis of femoral head. What is more, its advantages in rigidity extend its application in phalloplasty. Owing to the advancements in microsurgery, vascular compromise and flap failure have largely been avoided and many researches declare a low incidence rate of complication both on recipient site and donor site. However, complication like stress fracture and non-union could fail the rebuilding process, and donor-site morbidities like valgus ankle deformity or great-toe flexing contracture could lead to impaired walking ability and low-quality life. This review is aimed to summarize complication in types, incidence rates, and contributing factors of complication on donor and recipient site, and meanwhile update the measurement to quantize complication, technical refinement to avoid the complication. To the best of our knowledge, this is the first review focusing on complication both of donor and recipient area, covering all kinds of osteo reconstruction (extremities, mandible, spine and penile) by free vascularized fibular bone graft.

## Methods

Initially, a quick search was conducted by using term “(fibula [Title/Abstract]) AND (((flap [Title/Abstract]) OR (transfer [Title/Abstract])) OR (graft [Title/Abstract])) AND (review [Filter] OR systematic review [Filter])” on Pubmed to identify the complication types relevant to free vascularized fibular bone flap or fibular chimeric flap which has a bone component. In a generalized sense, the applied range of fibula bone flap may be roughly divided into four categories: extremities, spine, mandible and penis. However, according to the retrieved result-180 search results on fibular flap transfer, we found that the femoral head and the special condition of extremity reconstruction which necessitates a proximal fibular epiphyseal transfer are usually studied as isolated unit in published literatures. In fact, the purpose of hip-preserving treatment for femoral head necrosis is to introduce a new and stable blood supply, which is slightly different from that of repairing a segmental bone defect on extremities. In addition, the objective of proximal fibular epiphyseal transfer is to rebuild a near-nature growth potentiality for immature bone, or to reconstruct the spherical joint head (hip or shoulder joint) with the spherical fibular head. Both the reconstructive goal and the flap design have particularity. Therefore, we classified the applied ranges of FVFBG more detailedly into six categories: extremities, proximal fibular epiphyseal transfer, mandible, spine, osteonecrosis of femoral head, and penile. Accordingly, the complications on different reconstructive area were searched once again on Pubmed and studied in different separated section, such as “(extremit [Title/Abstract]) AND ((fibula [Title/Abstract]) AND (((flap [Title/Abstract]) OR (transfer [Title/Abstract])) OR (graft [Title/Abstract])))”. For the retrieval of donor site complications, we performed a separate search and excluded the fibular flap without bone component. Generally speaking, this is a Pubmed-based narrative review aimed to summarize all the potential risk involved in reconstruction by free vascularized fibular bone transfer. Evidences from systemic review, meta-analysis, and large-scale studies have been meticulously studied and updated if necessary. Certain emphasis is put on the highly-cited literatures. Sporadic cases are only adopted when uncommon complication is discussed, and would be presented as a whole after generalized search.

### Donor-site morbidities

FVFBG has been reported with higher success rate [1], superior union rate and anti-collapse effectiveness [2] compared with the non-vascularized fibular graft. However, according to one study, FVFBG increased the donor-site morbidities after graft harvest, which are attributed to loss of a pair of vessels leading to suppressed bone regeneration [3].

Wound healing problems in fibular flap donor areas, such as infection, bleeding, and delayed healing, are common early complications. But usually these do not affect the functional outcome, and will not cause long-term problems [4]. As long as the asepsis principle and skillful harvest have been ensured, these problems can mostly be prevented. Sensation problem (sensory loss, high sensitivity and pain) can be troublesome to patients, but most of them are recoverable and generally do not need surgical intervention. However, functional problems can lead to mild or severe deformity, and impair the balancing, walking or jumping abilities, which is the most concerned complication in the previous literature. Hence, the following will focus on functional loss, its surgical prevention and quantitative evaluation.

Surgical detail is significantly related to the donor-site function. Approach to harvest is the first consideration: Medial approach refers to a surgical alternative by which the fibular graft was elevated from the medial side. Technically speaking, lateral approach is more convenient in clinical practice because the fibular bone lies on lateral side just beneath the skin. But a comparative study by Philip [5] showed that medial approach led to less functional impairment of foot and ankle than lateral approach, with less neurologic and vascular impairment (totally 34 in 23 patients versus 46 in 19 patients) and higher American Orthopedic Foot& Ankle Society (AOFAS) score (94.4 points versus 85.6 points). Nevertheless, the medial approach is not applicable when a skin paddle or fibular head (epiphysis) is needed for the reconstruction, whose harvest is achievable only by lateral approach. A longer harvested fibula and longer operation time were important risk factors for morbidity. A possible explanation might be that damage increases with the length of the harvested fibula; alternatively, the longer operation time could be related to the difficulty of the dissection and the authors’ limited experience of fibula flap transfers, resulting in larger soft tissue damage during the operation [6].

Preoperative angiography of both legs should be carried out routinely to specify the distribution of blood vessels in the donor area. Anatomic variations where peroneal artery is absent or is the dominant artery supplying the leg and foot (known as peroneal arteria magna) should be detected in advance, otherwise harvest failure or ischemia of the distal limb would happen [7]. Preoperative angiography is more necessary for patients with comorbidities. In a 176-patient (mean age, 66.7 years) study by Alireza [8], 171 patients had at least 1 affected vessel and warranted further examination if a FVFBG was the planned option, and 30 patients had significant stenosis or complete occlusion of all vessels. High blood cholesterol, coronary heart disease and age are all significant contributing factors to vascular pathology.

Valgus ankle deformity happens particularly where there has been no fibular transfixion or where too little residual fibular bone has been left. In skeletally immature patients, the remaining distal fibula should be at least 6 cm or 10 cm [9] long and should be fixed to the distal fragment of the tibia with a transfixing cortical screw to prevent valgus deformity. Special care should be taken to preserve flexor hallucis longus, as the peroneal vessels are lying just posteriorly [10]. To avoid compartment syndrome, the closure of deep fascia should not be too tightly or in some cases should not be made [10].

Flexion contracture of the great toe is caused by the contracture and a tenodesis effect of the long-toe flexor tendons due to loss of muscle origins after fibular graft removal. Biomechanical model [11] showed that distal fibular harvest, including the separation of interosseous membrane can cause displacement of the extensor hallucis longus muscle and to a lesser extent, extensor digitorum longus muscle. Extending the osteotomy to proximal 2/3 would reduce cumulative displacement on the muscle’s origin and thus to minimize strength loss of dorsal-flexion on toes. Early toe-stretching exercise is suggested especially for patients who were found to have decreased motion in the early postoperative period. Or worst if it happened, a symptomatic patient would still benefits from flexor hailucis longus z-lengthening procedures [12]. Home-based exercise by climbing stairs or walking for at least 20 min (once daily, 3–5 days per week) at a comfortable speed could improve the strength of ankle dorsiflexion and foot eversion of the donor leg [13]. In general, the donor-site complication could be avoided, and preoperative vascular examination, careful dissection and appropriate post-operation exercise would give a help [14].

Since 1988 [15], measurement of function loss after fibula-resection has gained a lot accuracy in last few years. A significant reduction is found in maximum peak power per body mass (MPP), which is a decisive parameter for the realization of daily life, for example, getting up from a sitting position or climbing stairs. A loss of 22% of jumping height and 15% of take-off velocity further amplifies this point. Subjective balance testing reveals that 40.7% of the patients had postoperative gait insecurity or fear of falling [16]. Lots of study has been devoted to quantitive gait study to measure parameters based on strength and the time needed in walking, and the control group could either be the same limb before operation [17, 18], the healthy (contralateral side) limb [19], or least convincingly the other patients [20–22]. Stair ascent and descent has been used [22] and found that stride time, percentage of swing, and support phases did not differ among healthy and operated limbs and control subjects. But during stair descent, the patients had significantly larger pelvis inclinations than the control subjects. Between pre-operation and post-operation, the differences have been found both in bilateral evaluation of stance phases and swing, and unilateral double support time (pre 28.1%, post 26.3%, *p* = 0.05) which are an important phase in a gait cycle [23]. It has also been found that the impairment is under rehabilitation which necessitates adequate follow-up time. Lees [18] found that 1 month postoperatively, a significant (*p* < 0.05) decrease in the time-distance parameter and the peak plantarflexion, and dynamic range was seen. But at 3 months postoperatively only peak plantarflexion in swing was significantly (*p* < 0.05) decreased [18]. Completely insignificant results were also been found in some study [19, 24]. Generally speaking, the function impairment is minor and large-scale study with long time follow-up should be administrated to discover the donor-site function impairment which not only quantize the complication but also may indicate when and how should the post-operation exercise be administrated.

Incidence rate of function loss is confusing. In spite of these small-sized study in which evidence is not so convincing, and whether they are the different or the same reconstruction sites (extremities, mandible, or spine), the donor-site complication has widely-ranged incidence rates. In a prospective study with 157 Consecutive Patients, Adeyiza [25] found a low incidence of leg weakness (8%), ankle instability (4%), great toe contracture (9%), and decreased ankle mobility (12%). Sieg [4] reported in 62 donor sites in 57 patients who experienced fibular transfer with or without skin or muscle component, 30 patients (48%) had sensory deficits of calf and toes, 13 (22.8%) donor sites had prolonged wound healing, and 17 (29.8%) had hammer and claw toes and deficits in dorsal extension of the hallux, six (10%) had poor ankle function results according to Kitaoka ankle–hindfoot score*.* Ling [26] reported a incidence rate of chronic pain (6.5%); considerable gait abnormality (3.9%); ankle instability (5.8%); limited range of motion in the ankle (11.58%); reduced muscle strength (4.08%); claw toe (6.18%); dorsiflexion of the great toe (3.68%); and sensory deficit (6.958%). But generally speaking, the overall incidence rate of complication is still acceptable, and severe complication is rarely reported. Most of the donor-site complications are etiologically related to the surgical technique. Careful preoperative planning and meticulous dissection may eliminate the occurrence of these events [10].

### Complication on reconstructed area

Complication on reconstructed area can be classified into three types: one is about graft survival and usually happened at early stage. Another type is about incomplete reconstruction objective, for example, persistent malformation, repressed bone growth, delayed or unachieved bone union, stress fracture. The third type is recurrence of infection, tumor and radioactive ulcer which completely comes from problematic reconstructive area itself and is absent in reconstruction of congenital deformity. Vascular compromise is first noteworthy complication which could completely leads to transplantation failure. However, the common flap-monitoring methods like SPECT/CT [27], scintigraphy [28], intraosseous microdialysis [29] are indirect approaches which use the evidence of bone viability to identify the graft survivorship. It means these methods cannot directly reflect the quality of vascular anastomosis, let alone in a real-time manner. Doppler probe can provide a dynamic monitoring on the anatomical area which directly reflects the blood perfusion [30]. However, current reports of this technique mainly focus on the buried fascio-cutaneous flap in the reconstruction of head, neck and breast, but seldomly on osteo reconstruction. A skin window could be utilized as an option, but it is not applicable in spinal reconstruction which has multiple vital structure within the surgical field (lung, heart, aorta), or restoration of avascular of femoral head where the surgical field is too deep. Thrombosis and uncontrolled bleeding are also common complication due to fault anastomosis and easily affected by learning curve. Other recipient-site complication will be discussed in detail at seperate division.

### Reconstruction on extremity

The vascularity of the graft, which depends directly on the vascular anastomosis, has been described as a major factor influencing the outcome after FVFBG [31]. However, even flap monitoring is achievable on extremity but not always adequate for evaluating overall flap viability [32]. Fracture and delayed union are the most common complication. The stress fracture of the transferred fibula is a common complication with the reported rates ranging between 7.7% [33] and 35% [34], and often conservative treatment is recommended. Reported delayed union rates are ranging between 14% [33] -45% [35] in lower extremity and 20% [36]-45% [37] in upper extremity. Predisposing factors include inadequate fixation, infection, compromised vascularity of the graft, and inadequate preparation of the recipient site. Chemo- or radiotherapy do not contribute to nonunion [38]. In pediatric osteo reconstruction, it is noteworthy that even the defect has been reconstructed, the limb discrepancy could also happen due to the suppressive graft length-growth [39, 40]. For larger defects, division of a single fibula will not provide adequate length for double barrel grafting. Actually, single FVFBG has a higher incidence of stress fracture compared with double or dual FVFBG [41]. In those reconstruction with excessive length and width like femur and proximal tibial, double fibulas from the same patient should be advocated. Fibular graft combined with intercalary allograft, which was once a widely accepted reconstructive method, has been abandoned for a high incidence of nonunion, fracture, and infection [42]. Hypotrophy add a potential risk to stress fracture and happen as a lack of physiological loading of the graft. However, unlike mandibular reconstruction which has quantitative measurement, hypotrophy is poor documented in limb reconstruction.

### Proximal fibular epiphyseal transfer

Unlike the common vascularized fibular graft, the blood supply of vascularized fibular epiphyseal (VFG) transfer does not always come from fibular artery. Actually, the pedicle harvest has been provided with five different choice in chronological order: peroneal vessel [43], bi-pedicled transfer [44], single anterior tibial artery (antegrade [45] or reverse-flow [46]) and most-recently inferior lateral genicular vessels [47]. Anterior tibial artery is the most common choice and a reverse-flow design was reported with the best bone growth potential and low complication. But with the evidence of 4 clinical case reports of reconstructing Bayne and Klug Type III Radial Longitudinal Deficiency by Yang [47], and a 28-cadaver research [48] on blood supply of fibular epiphysis, the inferior lateral genicular was found to be an ideal alternative which has abundant anastomosis with anterior tibial artery, and a more donor-site-friendly choice because the harvest is well exempted from the transection of branches of the deep peroneal nerves, hence to lower the risk of sensory deficits on donor site.

VFG has been used to reconstruct a lost growth plate in the skeletally immature subject and articular surfaces. David [49] reviewed 62 patients from 20 publication and summarized that the most common complication in limb reconstruction by utilizing fibular epiphysis is fracture (35%). Full functional recovery seems more achievable in lower extremity reconstruction compared with upper limb (46% vs 25%). But as a lack of cases in lower limb reconstruction, the statistics was not so convincing. With the exception of Innocenti [43] who included 24 patients in his study, all other studies included less than 8 patients.

The common complications in the radius reconstruction are radial deviation and wrist subluxation, which often necessitate second surgery to correct or stabilize. Salah [50] reviewed 25 patients who underwent distal radius reconstruction, in which 16 patients developed recipient site complication, among which 4 patients had premature growth plate closure, 6 patient had radial deviation, and 4 patient had ulnar deviation or wrist subluxation.

The cases of using vascularized fibular epiphysis to reconstruct the lower extremity is small and the effectiveness is dissatisfactory. There were overall only 4 cases about hip [51, 52], 3 about the femur [53–55] and 3 case of lower leg [49]. The result of fibular epiphyseal transfer in hip articular reconstruction was the worst, while only one successful case (out of 4 cases) has been reported [51]. When the fibula head is embedded in the acetabulum, it becomes wider and thicker, which is close to the femoral head in morphology, which indicates the good plasticity of fibular proximal [51]. However, Other three cases were not so lucky. Among the three patients [52], one developed a progressive and painful stiffening, one had low graft growth and low acetabular filling index, and another one had slipped capital epiphysis and persisting hip instability. Possible reason lies the high stress-bearing due to standing position, the difficulty in articulation reconstruction and the inferior osteogenesis environment of infection. Reconstructions on femur or tibial are comparatively successful. But it should be noted that the diameter of fibula is not commensurate with that of femur. To provide a diameter-match, Fabio [53] introduced a double-barrel vascularized fibular graft for femur reconstruction, and both grafts experienced significant diameter (200%) and length growth (15%).

Overall speaking, this technique still leaves much to be desired for its high complication rate. However, the fibular head provides a good growth potential and its sphericityis close to the shape of humeral head and femoral head. At present, it is the most suitable choice for the repairing bone defects on immature skeleton, and the only autograft which could reshape into an articular head.

### Mandibular reconstruction

Fibular graft is the workhorse in mandibular reconstruction [56], for its strength of minimizing infection, resist absorption and allowing a simultaneous implant insertion, making the non-vascularized fibular graft a history. Brown [57] made a systemic review of 9499 mandibular defect treated by 6178 fibular, 1380 iliac crest, 709 scapular, 63 serratus anterior and rib, 32 metatarsal, and 10 humerus. In this review, the fibular graft was reported with the highest rate of second osteotomy (1.3% vs Scapula 0.43%), but a lower non-union rate (3.9% vs 9.1% radial forearm and scapular flaps), and lowest orocutaneous fistulas rate (4.9% vs iliac crest 7.8%). However, the evidence is low due to the under-reported fundamental outcomes.

Radiological change of non-union and bone absorption of fibular graft has been well documented and compared with those of other flap types [58]. Transferred fibular bones in the lower extremity tend to undergo hypertrophy, whereas in mandibular reconstruction 68% of them tend to be absorbed. Most of absorptions do not cause morpho-function problem, while severe absorption or atrophy may cause stress fracture or contour loss, and limit placement of a dental implant. Takaya [59] reported 13 patients out of 19 patients (69%) experienced fibular bone height decrease. The height decrease was more than 20% in 6 cases with a maximum of 38%, however, no stress fracture and bony non-union was observed. Takaya also concluded that it is preventable by ensure a direct nutrient artery to bone marrow, avoidance of osteotomies, and delayed placement of osseo-integrated dental implants. Tujia [60] found a statistically significant reduction in mandibular and fibular graft height, and 20% non-union rate, while no patients had an indication for reduction implant requirements or cosmesis. In fact, compared with other bone flaps, the risk of osteo-atrophy and resorption of vascularized fibula flap is comparatively low [61]. In a 3D volume analysis by Tommy [58], fibular has been demonstrated with a least bone resorption volume (1%) compared with other two popular graft candidate-iliac crest (3%) and scapular (14%). Age had no significant effect on bone resorption, but in older patients, bone resorption was more pronounced in female than in male [62]. The native mandible (especially edentulous) should also be concerned, because it was more absorbed than the fibula flap [63].

Just like the limbs, to assure a stable mechanical support to large defect, two-section or bilateral fibular grafts should be administrated in mandible reconstruction when necessary. In pediatric patients, impaired mandible growth after the reconstruction with fibular growth possible leads to malocclusion and facial asymmetry [64]. Luckily, a preservation of condyle, the osteo-growth center of mandible, has evidently deceased the risk of impaired growth from 50 to 18.5% [65]. Reconstruction during rapid growth period and a benign lesion characteristic facilitate the growth potential, while postoperative radiotherapy inhibits [66]. Worst, when the growth inhibition does occur, a further FVFBG can be transplanted again in the growing period, or orthodontic treatment be performed again in the mature period. However, vascularized fibular epiphysis has never been tried to increase the growth potential as a precaution.

Old age does not increase the risk of donor site complication or flap failure [67], but it does indicate a higher systemic complication and more difficulty in masticatory recovery. Sugiura [68] reported 9 out of 17 elderly patients (> 80 years old) had a systemic complication, in which delirium is the most common type. Peter reported age > 80 is an independent predictor of cardiopulmonary (52%) and neurological complication on multivariate analysis [69].

Complication relevant to plate system, which are used to stabilize the fibular graft, has also been debated. The evolution in type of metal, the size and malleability of the plates, and the locking mechanism has lower down the risk of complication like plate exposure, plate fracture, infection, screw loosening and worst surgical removal. Mandible plate, locking plate and reconstruction plate have been applied in mandibular reconstruction to fix the fibular graft, and mini-plate has gained an increasing use and currently become the dominant one, for its lower profiles and smaller screw diameters, which minimizing the risk of plate exposure [70, 71]. However, the mini-plate has also been suspected of less strength thus to cause fracture and screw loosening. Moreover, the mini-plate was more sensitive to varied loads than the reconstruction plate, and has less flexibility to absorb external forces. Mini-plates also caused high strain values, indicating hypertrophy risk in bone around the screw holes [72]. In a systemic review of 544 patients by Shang-Ping Liu [73], it found that when miniplates were used with fibular flaps there were 10.3% (56/544) complications, of which 4.8% (26/544) were loosening of the screws, 2.6% (14/544) fracture of the plate, 1.5% (8/544) exposure of the plate, and 6.4% (35/544) infection. There are also literatures that believe that there is no difference between miniplate and traditional plate in the incidence of complications [74], and also there is no difference between mini plate and biodegradable plate [70], so is the conclusion by meta-analysis [75]. Radiotherapy and diabetes increased the incidence rate of plate-related complication [69]. And FVFBG acts more as a risk factor [76, 77] than as a protective factor [78] compared with other bone graft type in the aspect of severe complication which need a surgical removal.

Vascular pedicle ossification refers to graft pedicle experiencing calcification which could be identified both on CT scanning and with naked eyes under operation. Though it has a low incidence (4.4%) [79] and only reported as sporadic cases for 12 times [80–83], it causes hard swelling, trismus, and severe pain which absolutely indicate a surgical intervention. Interestingly, all these cases are documented in mandibular reconstruction and no cases in other reconstruction like spine or extremities Potential causes of this complication include bony contact, stress, infection and steroid-use. To avoid these, an extra resection of periosteum along the vascular pedicle during flap harvesting is recommended. Or if the calcification inevitably happened after the operation, the removal of heterotopic ossification along the pedicle could still be operated at the second stage [79].

Computer-aided design and manufacturing (CAD/CAM) technology [84] in mandibular reconstruction has been reported with higher accuracy which allowed better functional outcome [85]. The short and flat morphologic characteristics of fibular graft does not match well with the unique and three-dimensional parabolic shape of the mandible. The mismatch leads to vertical discrepancy, which necessitates long abutments, from 7 to 10 mm in some cases, and consequently limits the choice of the further implant system. This donor-recipient configural mismatch brings about the enthusiasm about this virtual reality technique. However, the accuracy of CAD/CAM is more achievable in planned cutting osteotomy on mandibular [86, 87], which would benefits the preservation of condyle. But parameters like contour and position of plate, or dental implant mandibular segmental osteotomy of fibular, has not been completed in the CAD/CAM system [88, 89]. The computer-aided surgery is a still a limited and under-developing tool in mandibular reconstruction, and for beginner traditional method is more suitable.

### Spinal reconstruction

Fibular graft is indicated when there are complex primary or secondary defects and/or loss of structural integrity of one or more spinal segments that require adequate, long-term, mechanical stability. The application of fibular flap in spine reconstruction is based on the success of limb reconstruction. As a result, the developmental history is short and only sporadic cases are available, the documented complications of spine reconstruction are not as complete as the that in extremities.

Non-union is the most-common complication and has been reported with a incidence rate of 4% [90], 6.3% [91] and 14% [92, 93]. Vascularized fibular transfer has evidently decreased the risk of graft nonunion compared with non-vascularized fibular graft [94]. But still, it happened and might result from vascular complication [92]. To deal with the potential risk of thrombosis, blood leakage and flap failure, a suitable approach for exposure, a successful anastomosis and an appropriate acceptor vessel should be achieved [95]. Segmental renal artery can be selected as one of the recipient vessels in spinal reconstruction surgery without detrimental effect on renal function [96], but the superior and inferior mesenteric vessels is not recommended for potential risk of intestinal ischemia and necrosis [95]. If no local acceptor vessels can be found, there is a possibility to construct an arteriovenous loop from the greater saphenous vein [97]. Dissection of recipient vessel should be prepared during flap harvest and before transfer, because the extensive period of time between the dissection of the fibular flap and the actual transfer can produce deep venous thrombosis on donor-site [90].

To ensure an adequate mechanical support to avoid fracture and collapse, the fibular should be double-, triple- or quadruple-barrel construct if necessary. But when multi-barrel fibular grafts are applied, it should be realized that there is a risk of pushing the most posterior fibular strut into the direction of the spinal cord [90]. Another aspect of complication is relevant to the spinal surgery itself instead of fibular transfer. The accidental injury of surrounding tissue by accident might impair artery (aorta, vertebral artery and subclavian artery), esophagus and lung which may lead to massive haemorrhage, pneumothorax or pulmonary embolism [90, 98].

### Revascularization of Osteonecrotic femoral head (ONFH)

Vascularized grafts can be used in the treatment of osteonecrosis at a variety of anatomical sites, but most commonly used in young patients with ONFH. In a systemic review of 1270 procedure at an average of 8.3 years [12], 215 complication (16.9%) was observed including 146 (11.5%) and 69 (5.4%) were referable to the donor and graft sites, respectively. Sounding high at the first glance at the overall complication, only 54 patients (4.3%) should count as major complications or experience chronic pain which required additional procedure including hardware removal, tendon lengthening, removal of heterotopic bone, sensory repair and so on. Pin migration (overall 2.4%, and accounting for 43% of graft-site complication) and heterotopic ossification [98] (32%) are the dominate complication in graft site. Usually they are asymptomatic and diagnosed by radiographic evidence, but severe tenderness and discomfort would indicate a surgical removal. Femoral neck is transiently weakened secondary to removal of bone core to allow insertion of fibular graft, which result in the possible risk of though infrequently-happening but troublesome fracture. The risk is high in the early stage, but with the standardization of postoperative management (6 weeks of weight-bearing prohibition on the operation side), the previous complications of up to 20% [99] gradually fade out of the orthopedic doctor’s vision (0.7%) [100].

The incidence of conversion to total hip arthroplasty and radiographic necrosis progress are major parameter studied in literatures. Not included as complications though, they are definitely surgical failure to arrest necrosis progression. Risk factors for reconstructive failure or graft absorption has been routinely studied, like age, sex, different predisposition (steroid-use, trauma, alcohol-abusing) and different disease stage. However, as the bone flap survivorship is monitored indirectly by radiography (usually SPECT/CT and scintigraphy) and based on bone viability, there is an absence of key evidence for the vascular-anastomosis quality. Once this confounding viable-the quality of vascular anastomosis is not controlled. The true relationship between the risk factors and reconstructive failure will be deviated. Histopathological analysis should be the most convincing examination but is limited by small size [101, 102] . However, at least, it is clear that the postoperative steroid-use is a risk factor for the progression of osteonecrosis [103, 104].

Compared with non-vascularized fibular graft and core compression, the vascularized fibular graft is a superior choice for lower rate of conversion to THA or radiographic necrosis progress [105]. But no sufficient statistics to compare fibular versus iliac flap. The only evidence is in a comparative study which recorded insignificant difference in therapeutic effectiveness [106]. Donor site morbidity cannot be compared between iliac and fibular graft, because they are harvested from different area. But theoretically and practically, partial support loss on lateral ankle due to fibular flap harvest may impair walking or jumping ability. Even if the risk is low, it is not as safe as the anterior superior iliac spine, which does not involve with weight-bearing problem.

Recipient vessel and surgical approach have also been studied. Different recipient vessels [107] (three kinds) do not affect the incidence rate of complication related to hip reconstruction (including conversion rate and radiographic progression) and vascular anastomosis. Anterior approach [108] for transplanting fibular into femoral head has been recorded in 578-case study with low complication, in which only one deep infection, 11 temporary sensory loss on thigh, and 9 restricted motion of great toe. The benefits lie in a broader exposure of recipient vessel with sufficient length and size, a thorough debridement of necrotic bone and a direct visualization permitting reliable anastomosis. However, the study had no traditional lateral approach as a control group.

### Penile reconstruction

In previous-published literature of 1196 patients under phalloplasty, only 141 patients were operated with fibula osteo-cutaneous flap including 5 without osteo component, which is secondary to the workhorse-the radial forearm flap [109]. Advantages of fibular osteo-cutaneous flap is a bony component which provide long-term rigidity allowing deep penetration during sexual intercourse, and decrease the risk of cutaneous erosions. However, dyspareunia might be complained by their sexual partner due to lack of malleability. If happened coincidentally, a modification by creating pseudo-joints with segmental osteotomies and fascia interposition would alleviate the pain [110]. To ensure a sensibility on neophallus, sural nerve should be harvested and then coaptated to dorsal penile nerve. Nevertheless, a comparative study shows that patients who underwent the fibular flap experienced worse sensibility than forearm flap [111]. Possible reason lies in the inconstant anatomy of lateral sural cutaneous nerve which need scrupulous dissection, and one less nerve is coaptated in the fibular transfer. The most-commonly-seen complication is urethral stricture (stenosis) (17%), followed by partial flap loss/distal necrosis (11.9%), urethral fistula (9.6%) and complete flap loss (1.5%), all of which are also recorded in phalloplasty by other free tissue transfer [109]. Additional operation is often needed to treat the urethral complication-surgical closure is needed for 67% of patients with a fistula, while surgical expansion is needed for 68.1% of patients with a stricture [110, 112]. For prevention of fistula, pars fixa should be lengthened to clitoris by using the anterior vagina flap [112]. Bone absorption or fracture has never been reported in either fibular or other graft. Actually, a long-term radiographic study shows the mineral density values in neophallus is closed to that on residual fibula [113]. Generally speaking, the fibular osteo-subcutaneous graft is a satisfying alternative to forearm flap due to the similar complication rate. Further technical refinement and standard procedure are expected in the future.

## Conclusions

In summary, the complication of osteo reconstruction by vascularized fibular is acceptable and need further improvement. Technical tips on flap harvest and transplantation have been provided to lower down the risk of vascular compromise and other complications. Computer assistance is still under exploration and might be good option to avoid mismatch of graft, implant, and mandibular residual. Proximal fibular epiphyseal transfer is more challengeable than a diaphyseal transfer and further basic research is needed to ensure the growth of transplanted fibular proximal adjustable to the recipient site. Current monitoring devices on fibular flap survivorship is mainly rely on indirect approaches like SPECT/CT, scintigraphy, intraosseous microdialysis and so on. We are looking forward to more direct and real-time monitoring device on fibular flap perfusion. Dynamic measurement of functional loss on donor-site needs the large-scale research to quantize the complication and guide gradual weight-bearing.

## Data Availability

The datasets used and analysed during the current study available from the corresponding author on reasonable request.

## Abbreviations

FVFBN: Free vascularized fibular bone graft
VFG: Vascularized fibular epiphyseal
CAD/CAM: Computer-aided design and manufacturing
ONFH: Osteonecrosis of femoral head
